# Proximal determinants of suboptimal early child development during the first three years of life in socially deprived Mexican contexts

**DOI:** 10.1371/journal.pone.0291300

**Published:** 2023-11-02

**Authors:** Edson Serván-Mori, Amado D. Quezada-Sánchez, Evelyn Fuentes-Rivera, Carlos Pineda-Antunez, María del Carmen Hernández-Chávez, Angélica García-Martínez, Raquel García-Feregrino, Abby Madrigal, Bárbara Guerrero, Gerónimo Medrano, Lourdes Schnaas

**Affiliations:** 1 Center for Health Systems Research, National Institute of Public Health, Cuernavaca, Morelos, Mexico; 2 Center for Evaluation and Surveys Research, National Institute of Public Health, Cuernavaca, Morelos, Mexico; 3 Center for Demographic, Urban and Environmental Studies, El Colegio de Mexico, Mexico City, Mexico; 4 Department of Developmental Neurobiology, National Institute of Perinatology Isidro Espinosa de los Reyes, Mexico City, Mexico; 5 Lucy Family Institute for Data and Society, University of Notre Dame, Notre Dame, IN, United States of America; 6 Integral Services for Childhood Attention Consulting, Mexico City, Mexico; Aga Khan University Hospital, PAKISTAN

## Abstract

Assessing the status and determinants of early child development (ECD) requires accurate and regularly updated measurements. Yet, little information has been published on the subject in low- and middle-income countries, particularly regarding the proximal determinants of childhood development in contexts of high social marginalization. This article analyzes the factors that favor or mitigate suboptimal ECD outcomes in Mexico. A cross-sectional study was conducted using recently collected data for 918 children aged 0–38 months from socially marginalized communities in 23 Mexican municipalities. The ECD outcomes of the children were estimated based on indicators of chronic undernutrition and neurodevelopment (normal, lagging and at risk of delay). The distribution of outcomes was described across the ECD proximal determinants analyzed, including the co-occurrence of chronic undernutrition and suboptimal neurodevelopment. Covariate-adjusted prevalence of the ECD outcomes and co-occurrences were calculated as post-estimations from a multiple multinomial logistic regression. The prevalence of chronic undernutrition was 23.5%; 45.9% of children were classified with neurodevelopmental lag, and 11% at risk of neurodevelopmental delay. The prevalence of stunting co-occurring with suboptimal neurodevelopment came to 15.4%. The results of the multinomial logistic regression model indicated that early gestational age, low birth weight, a low household socioeconomic level, being male and having numerous siblings were all associated with the co-occurrence of chronic undernutrition and suboptimal child neurodevelopment. This study identified important predictors of child development in the first three years of life, specifically in two of its principal indicators: nutritional and neurodevelopmental status. Most of the predictors observed can be improved by means of social programs and interventions.

**Trial registration:** ClinicalTrials.gov ID: NCT04210362.

## Introduction

Intervening and improving conditions of equity during the first three years of life offer a unique opportunity to shape the future of society [1, 2]. Suboptimal, or below-potential, development in girls and boys during those years impacts society as a whole [3]. The biological mechanisms by means of which delays in early child development (ECD) compromise the lifetime opportunities and accomplishments of individuals are well known [4].

Measuring ECD accurately and regularly is indispensable for assessing its status, understanding its determinants, and designing as well as implementing effective public policies in this regard [5]. However, low- and middle-income countries have published little information on ECD among those living in contexts of high social marginalization [6, 7]. The logistical and methodological challenges posed by the multidimensional nature of ECD are at least partially responsible for the dearth of available information on the subject [8, 9].

Children raised in unfavorable socio-environmental contexts are more likely to experience suboptimal ECD. Such environments are detrimental to the growth and development of the brain, permanently altering its functioning [1, 10]. Poor children face more adverse conditions and enjoy fewer learning opportunities–both common mechanisms of the poverty trap–than do their counterparts in less disadvantaged situations [11]. Moreover, deprivation carries long-term psychological consequences including epigenetic effects on brain and cognitive development [12].

**Fig 1:**
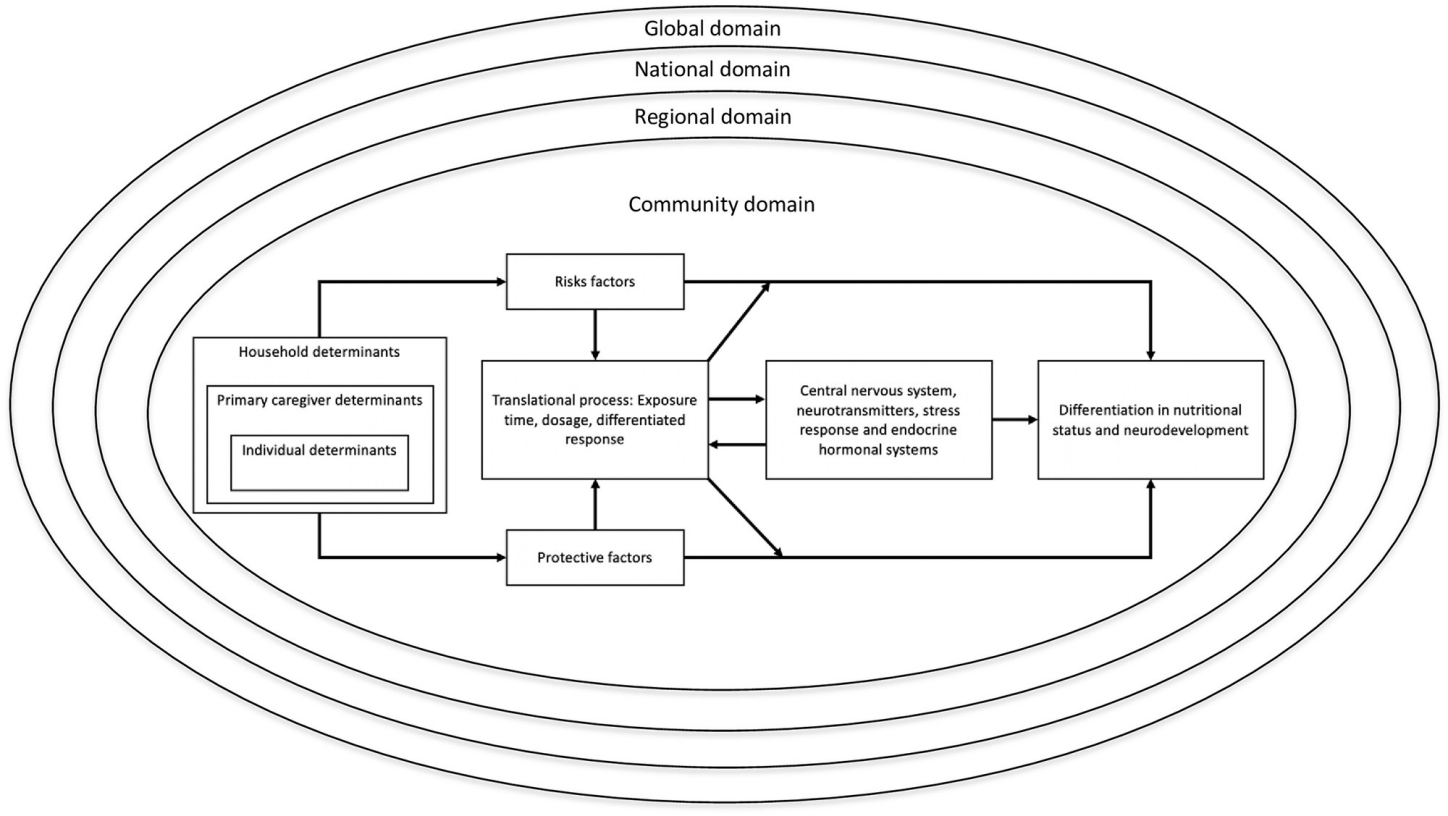
Conceptual scheme [17, 18].

ECD is directly related to socioeconomic level, food insecurity, stunting in low- and middle-income countries [6, 7, 13], breastfeeding [14], child-rearing practices [15], and other variables recognized as proximal determinants of child development. The term “proximal determinants” refers to social factors such as work status and other socioeconomic conditions shaping personal development, as well as to individual factors such as age, sex, and other biological characteristics that influence health and development [16]. Walker et al. [17] outlined the relationships that characterize the links between ECD and its proximal determinants. The first concerns family poverty, the root of both numerous risk factors and the lack of protective factors. Our framework, adapted from Walker et al., acknowledged that the household, primary caregiver, and individual determinants are the source of either risk or protective factors. Secondly, Walker et al. demonstrated that the amount and duration of, as well as differential reactivity to, exposure to risk/protective determinants (translational process) are reciprocally related to brain function and structure (the central nervous and endocrine systems, neurotransmitters, and stress response). Finally, the authors revealed that timing, dosage, and differential reactivity mitigate the effects of risk and protective factors upon child development. This conceptual framework has been complemented by other approaches including the Total Environment Assessment Model for Early Child Development (TEAM-ECD), advanced by Irwin et al. [18]. The latter introduced four additional levels of ECD determinants. According to these authors, the *community domain*, or environment where children live, determines their access either to resources that foster their growth (e.g., amenities) or to potential risks (e.g., pollution). The *regional domain*, which comprises degree of urbanization, population health status, and other socioeconomic as well as political factors, affects ECD through the influence of these variables on the family. The *national domain* impacts the environments that bear upon the wellbeing of children through policies and laws. And finally, the *global domain* affects ECD through its influence on national policy [18, 19]. Fig 1 presents the two models combined.

The effects of proximal determinants on the health of children, often with long-term consequences, have been widely recognized [20–24]. For instance, it has been shown that social and economic contexts explain 50% of the health status of populations, health systems contribute 25%, the physical environment 10%, and genetic as well as biological factors the remaining 15% [25]. A favorable ECD environment includes proximal environmental determinants that support the full development of girls and boys. Such determinants allow for meeting childhood needs regarding not only food, health and protection, but also affection, social interaction, communication, emotional safety, stability, and access to opportunities for exploring and discovering the world [26]. Each proximal determinant is causally linked to ECD through specific mechanisms. For instance, individual traits like age, sex, birth weight, and gestational age directly impact ECD, with normal birth weight promoting adequate nutrition and full-term births supporting healthy neurological development. Proximal determinants also apply to those closest to children, typically their primary caregivers, where higher educational attainment tends to enhance child-rearing practices. Additionally, caregiver characteristics like age and marital status also play a causal role in influencing ECD. Proximal determinants concerning the homes of the children impact ECD as well. Enjoying a higher socioeconomic status in the home allows for implementing better child-rearing practices. Growing up in an indigenous household can also constitute a determinant of child-rearing practices, with an impact on ECD. Finally, crowded living conditions and specific types of households can have repercussions for ECD.

Understanding the determinants of undernutrition and suboptimal neurodevelopment in contexts marked by high social vulnerability is essential for designing effective interventions [17]. With this in mind, we analyzed the factors that favor or mitigate suboptimal ECD outcomes, using data collected in 2019–2020 for children aged 0–38 months in Oaxaca, Mexico.

## Methods

### Design and participants

We conducted a cross-sectional analysis of primary data pertaining to children aged 0–38 months from marginalized communities in 23 municipalities in the state of Oaxaca, located in southwestern Mexico. With a population of approximately four million inhabitants (31% of them indigenous), Oaxaca is one of the Mexican states with the highest levels of social marginalization as well as conditions of below-national-average life expectancy at birth [27]. In 2018, 66% of the population in the state lived in poverty and 26.9% in extreme poverty, 27% suffered from educational gaps, and 77.9% lacked basic services at home; while only 15.9% had access to health services and 31.4% to food [28].

### Data collection and ethics

The individuals analyzed in this study participated in the baseline measurement impact evaluation of the Neurological and Psycho-affective Development Program (PDNyP by its Spanish initials) on child neurodevelopment (in progress) [29], designed and implemented by the Mexican non-governmental organization Un Kilo de Ayuda A.C. Details concerning the sample design and the PDNyP itself have been presented elsewhere [29].

During 2019 and 2020, anthropometric, health, sociodemographic, ECD, and family data were collected, as well as data on the immediate environment of the children. Local authorities facilitated the initial contact, aimed at identifying children of interest along with their caregivers, who were asked about their willingness to participate in the study. First contact with participants was established in two ways: first, authorities of the municipalities visited convened a meeting of the population of interest where the PDNyP and the activities that would be carried out as part of the research project were explained. Second, a census of the population in the selected municipalities was conducted using a Municipal Register provided by authorities. In households with children aged three years or younger, caregivers were queried about their intention to take part in the study. Once potential participants had been identified, the recruitment phase was conducted from July 2019 to March 2020. We obtained sample measurements for a total of 1,179 children. The present study focused on children whose mothers were their main caregivers (n = 1,073). Of these, 155 (14.4%) with missing values in any of our analytical variables were excluded. Our final sample size came to 918 children aged 0–38 months.

Participation within the studied population was voluntary, and we secured prior written consent from all participants. The research protocol was approved by the Ethics Committees on Research and Biosafety of the National Institute of Public Health in Mexico (CI-896-2018/1538) and is registered in ClinicalTrials.gov (CT/ID: NCT04210362). Data collection instruments were piloted and administered by means of electronic questionnaires on mobile devices with the Android^®^ operating system, designed as part of the REDCap (Research Electronic Data Capture) web application [30]. Data quality was guaranteed by applying auditable algorithms for the systematization and automatic identification of possible errors in the values of the measured characteristics.

### Measurement of suboptimal ECD

We analyzed ECD based on a joint assessment of the nutritional and neurodevelopmental status of each study participant. We evaluated child nutritional status using anthropometric measurements of length/height- and weight-for-age taken by trained and standardized personnel in accordance with international protocols [31, 32]. To measure length, we used a 200Kg capacity SECA brand electronic scale (874TM), with an accuracy of 50g< 150Kg >100g, and a 100cm long SECA brand plastic infantometer (416TM), with an accuracy of one millimeter; a 198cm long SECA brand plastic stadiometer (217TM) with an accuracy of one millimeter was used to measure height. Following WHO growth standards and their interpretation [33, 34], we established the following indicator for suboptimal physical development: chronic undernutrition (or stunting), defined as length- or height-for-age Z < -2 score.

We evaluated neurodevelopment based on the Child Development Evaluation Test, Second edition (EDI-II, by its acronym in Spanish) [35, 36], whose conceptual and analytical benefits have previously been noted [37]. This screening test, designed for the early detection of neurodevelopmental problems in children ranging in age from 1 month to 59 months and 29 days, has been validated for use in the Mexican population [38]. It consists of items aimed at 14 age groups, involving direct observation and neurological exploration of the children, targeted questions, and structured activities for evaluating development in the following areas: gross and fine motor skills, knowledge, biological risk factors, warning signals and symptoms or alarming signals.

The test conceptualizes the following development areas separately: gross motor skills, which include postural responses, cephalic balance, sitting posture, crawling and standing posture; fine motor skills, which refer to the movement of the hand when grasping or manipulating objects; language, referring to all gestural and linguistic forms of communication; social behavior, meaning personal reactions to the social culture in which the child lives; and knowledge (this section of the test, designed for children aged 37–59 months, explores the ability to draw on past experiences and apply them to new situations) [36]. EDI-II neurological exploration focuses on the motor system, with emphasis on muscle tone and reflexes [39]. Biological risk factors are defined as any situation experienced by a child that may predispose him/her to developmental delay. Warning signals refer to the presence of symptoms and signs of delay, or the failure to meet certain developmental milestones at specific ages, indicating the possibility of developmental problems. Warning signals are the clinical expression of a probable delay or deviation from the normal development pattern [36]. Test items consist of both direct observation of the child, as well as questions addressed to the child’s caregiver. The final test results are expressed in the form of a traffic-light scale. Classification criteria are age specific but can generally be described as follows:

- Normal development (Green). The child exhibited all the development milestones of the corresponding age group, and no warning signals or abnormal results were detected during the neurological examination.
- Developmental lag (Yellow). The child partially exhibited the development milestones of the corresponding age group but did show all the milestones of the preceding age group. Warning signals and biological risk factors might be present, but no alarming signals or abnormal results were detected during the neurological examination.
- At risk of development delay (Red). The child exhibited neither the development milestones of the corresponding age group nor those of the preceding age group; he or she showed alarming signals and/or abnormal results during the neurological examination.

Trained, standardized, and certified personnel administered the test; they also gathered and processed the results.

### Proximal determinants

In line with previous studies [6, 7, 11, 13, 14, 40], we analyzed three groups of ECD proximal determinants: (1) *individual*: age (<12, 12–23, and 24 or more months), sex (male = 1, female = 0), gestational age (28–36, 37–38, 39–40, and 41–43 weeks), low birth weight (<2.5kg), number of siblings (none, one, and two or more), and health-insurance coverage; (2) *mother*: age (15–24, 25–29 and 30 years or older), marital status (non-union: single, widowed/divorced/separated, or in union: married/free union), schooling (elementary or none, middle, and high school or higher), and work status during last week (working or not); and (3) *home of residence*: a nuclear-family household (a mother and father living with their children), an indigenous household (where the head of household, a spouse or a family member–a parent, grandparent or one more remote–was an indigenous-language speaker) [41], overcrowding (more than two occupants per room on average) [42], participating in a government social program, and socioeconomic status (SES), according to an index categorized into terciles (low, medium and high), which was constructed based on the first principal component of eleven variables pertaining to the ownership of assets, as well as housing materials and infrastructure. The first principal component absorbed 27% of total variation, with correlations between variables and the first principal component ranging from 0.34 to 0.62 [43].

### Statistical analysis

We first estimated the prevalence of stunting and neurodevelopmental status (normal, lagging and at risk of delay) with 95% confidence intervals. Height (or length) and age in days were plotted on a scatter diagram delineating the WHO growth standard median height (or length) with reference lines placed at ±2 and ±3 standard deviations from the median; males and females were analyzed separately. To assess the co-occurrence of chronic undernutrition, as measured by stunting and neurodevelopment outcomes from EDI-II, we defined a six-category outcome variable as follows: (1) non-stunted and normal neurodevelopment; (2) non-stunted with a neurodevelopment lag; (3) non-stunted and at risk of neurodevelopmental delay; (4) stunted with normal neurodevelopment; (5) stunted with a neurodevelopment lag; and (6) stunted and at risk of neurodevelopmental delay. We estimated the prevalence of each outcome category with their 95% confidence intervals. To describe the distribution of the proximal ECD determinants we used frequencies and percentages.

Next, we estimated a multiple multinomial logistic regression model using the six-category outcome variable described above as dependent variable and all the proximal ECD determinants as predictors. After estimating this model, we obtained covariate-adjusted prevalence for each outcome category through predictive margins. We then calculated covariate-adjusted prevalence for the following unions and specific categories of the outcome: (a) stunting (outcomes 4, 5 and 6); (b) neurodevelopment lag (outcomes 2 and 5); (c) at risk of neurodevelopment delay (outcomes 3 and 6); (d) neurodevelopment lag or at risk of delay (outcomes 2,3,5 and 6); (e) stunting with neurodevelopment lag (outcome 5); (f) stunting and at risk of neurodevelopment delay (outcome 6); and (g) stunting with neurodevelopment lag or at risk of delay (outcomes 5 and 6). We performed comparisons of covariate-adjusted prevalence between categories of each predictor using Wald tests, and set the statistical significance level at 0.05, 0.01 and 0.001. S1 File presents additional details of the multiple multinomial logistic regression model and the predictive margins obtained as post-estimations.

For estimating raw (covariate-unadjusted) prevalence we fitted a separate multinomial regression model for each proximal ECD determinant and calculated the seven prevalence for unions and specific categories of the outcome described above. All model standard errors were adjusted for data dependencies within municipalities [44]. Predictive-margin standard errors were obtained utilizing the Delta method [45], and analyses were performed using the Stata MP v17.0 statistical package [46].

## Results

### Distribution of stunting, neurodevelopment outcomes and their co-occurrence

**Fig 2:**
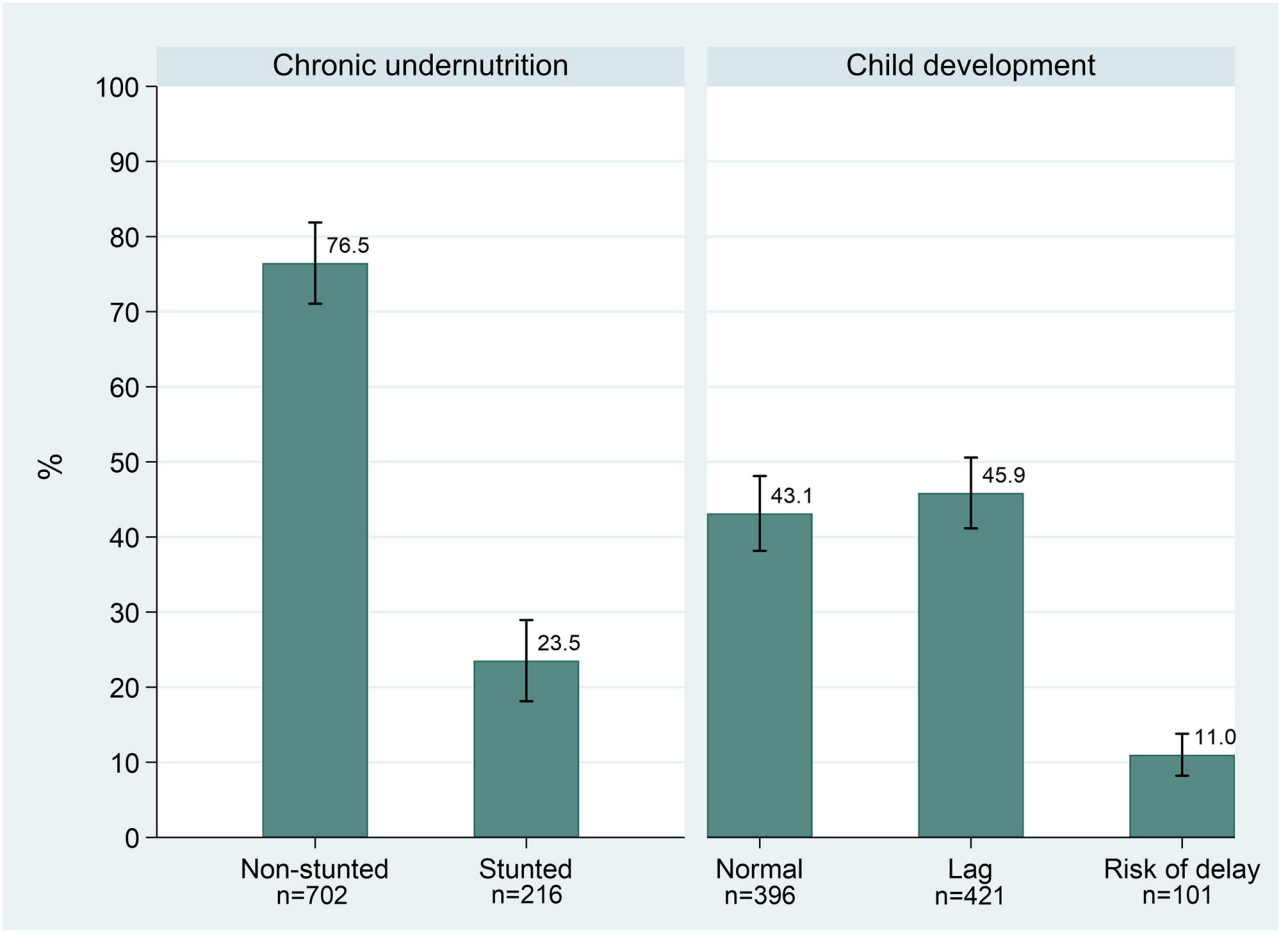
Distribution of chronic undernutrition status and neurodevelopment outcomes.

**Fig 3:**
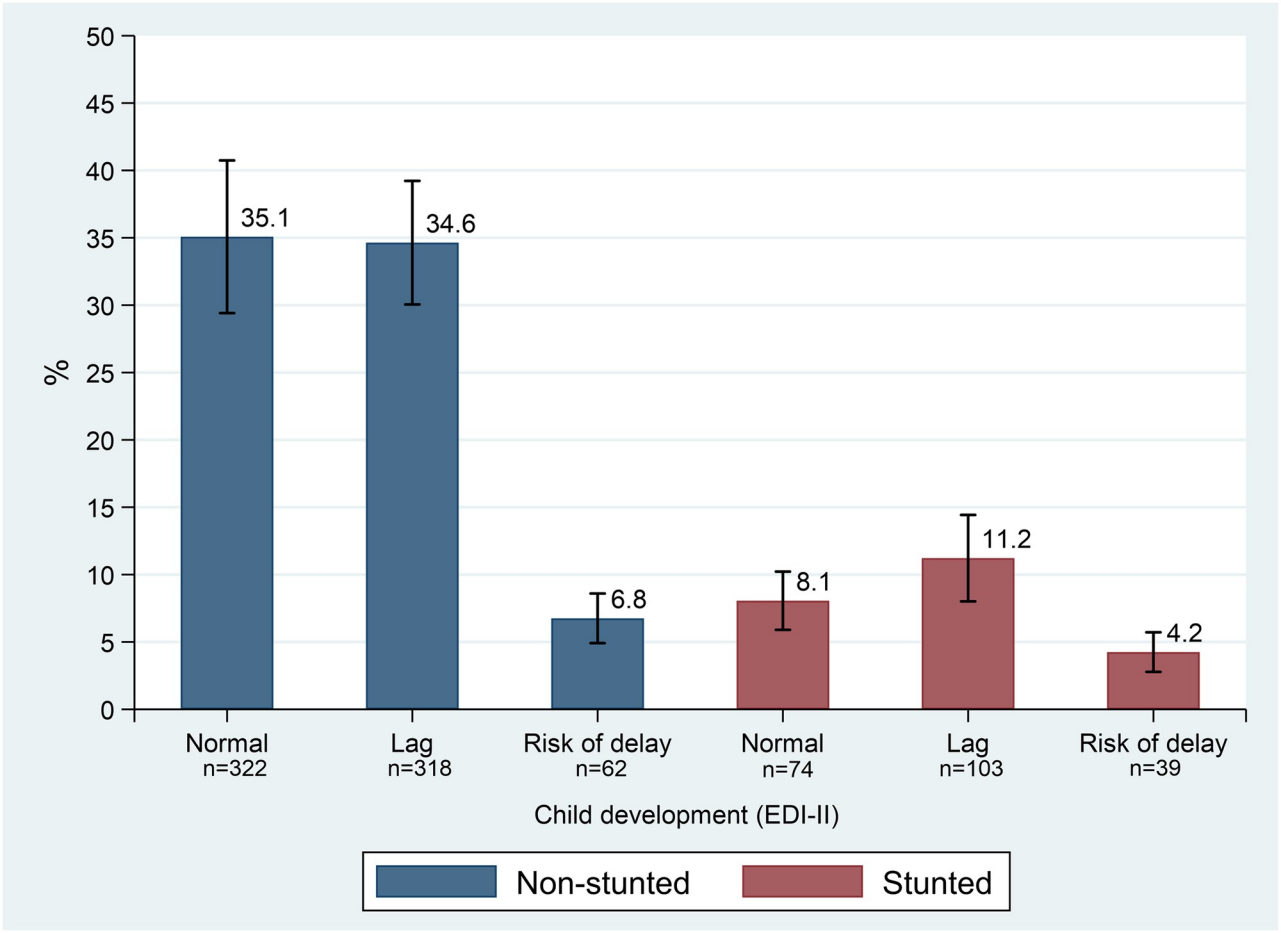
Joint distribution of chronic undernutrition status and neurodevelopment outcomes.

Our evaluation yielded the following results: 23.5% of children were stunted and 45.9% classified as having neurodevelopment lag, while 11% were at risk of delayed neurodevelopment (red light). Additionally, 11.2% experienced the co-occurrence of stunting and lagging neurodevelopment, and 4.2% were stunted and at risk of neurodevelopmental delay (Figs 2 & 3). The supplementary material (S1 Fig in S1 File) contains a scatter plot of length or height in centimeters vs. age in days, with most children in our study exhibiting a length or height below the WHO median standard.

### Distribution of ECD proximal determinants across the analytical sample

As to the analyzed proximal determinants of ECD (Table 1), 76% of the children were ≥1 year, 53% were male, 8% had been born prematurely, 11% suffered from low birth weight, 35% had no siblings, and 22% had no health-insurance coverage. With respect to mothers, 66% were <30 years old, 87% were married or living in union, 60% had secondary schooling or less, and 62% had not worked during the previous week. Statistics regarding household characteristics revealed that 84% of children lived in nuclear families, 29% in indigenous households, and 23% in overcrowded houses, while approximately 30% lived in homes with beneficiaries of social programs.

**Table 1:** Suboptimal ECD prevalence by categories of proximal determinants.

	n	%	Stunting, %	Neurodevelopment			Stunting and neurodevelopment		
	918	100.0		Lag, %	At risk of delay, %	Lag or risk of delay, %	Lag, %	At risk of delay, %	Lag or risk of delay, %
*Child characteristics*									
Age (months)									
< 12	217	23.6	21.7 ± 3.5	46.1 ± 5.2	18.4 ± 3.9~	64.5 ± 4.9~~	9.7 ± 2.0	6.0 ± 2.0	15.7 ± 2.7
12 to 23	380	41.4	25.3 ± 2.9	51.3 ± 3.2	7.4 ± 1.4*	58.7 ± 3.7	14.2 ± 2.5	2.6 ± 0.6	16.8 ± 2.7
24 to 38	321	35.0	22.7 ± 4.0	39.3 ± 2.3°°°	10.3 ± 2.0	49.5 ± 2.1°	8.7 ± 1.9°	5.0 ± 1.6	13.7 ± 2.7
Sex									
Male	486	52.9	25.5 ± 3.0	47.1 ± 2.6	13.8 ± 1.8	60.9 ± 2.5	12.6 ± 1.9	5.3 ± 0.9	17.9 ± 2.4
Female	432	47.1	21.3 ± 3.1	44.4 ± 2.6	7.9 ± 1.4***	52.3 ± 3.0***	9.7 ± 1.8	3.0 ± 0.9*	12.7 ± 2.3*
Gestational age (months)									
28 to 36	76	8.3	47.4 ± 7.6~~~	28.9 ± 4.3~~~	23.7 ± 4.6~	52.6 ± 3.4	13.2 ± 3.2	17.1 ± 3.9~~~	30.3 ± 4.9~~~
37 to 38	214	23.3	26.2 ± 2.7*´	50.9 ± 4.0***	8.4 ± 3.2**	59.3 ± 3.8	14.0 ± 3.3	4.7 ± 1.9***	18.7 ± 2.4
39 to 40	538	58.6	20.3 ± 3.8	45.7 ± 2.7	11.2 ± 1.4^^^	56.9 ± 3.1	9.9 ± 2.6	2.8 ± 0.7	12.6 ± 3.0
41 to 43	90	9.8	16.7 ± 3.7```	48.9 ± 6.3`	5.6 ± 1.8```	54.4 ± 5.7	11.1 ± 3.9	1.1 ± 0.9```	12.2 ± 3.6```
Low birth weight (<2500g)									
No	817	89.0	21.2 ± 2.1	46.6 ± 2.2	9.3 ± 1.2	55.9 ± 2.5	10.8 ± 1.5	2.7 ± 0.5	13.5 ± 1.7
Yes	101	11.0	42.6 ± 6.0***	39.6 ± 5.3	24.8 ± 4.7**	64.4 ± 3.5*	14.9 ± 3.2	16.8 ± 3.7***	31.7 ± 4.3***
Number of siblings									
None	324	35.3	21.0 ± 3.6~	44.4 ± 5.2	9.9 ± 2.4	54.3 ± 4.6	10.5 ± 1.9	4.3 ± 1.5	14.8 ± 2.7
One	358	39.0	22.3 ± 3.0	46.6 ± 3.5	10.6 ± 1.2	57.3 ± 3.6	10.9 ± 2.3	3.6 ± 0.7	14.5 ± 2.4
Two or more	236	25.7	28.8 ± 3.1°	46.6 ± 3.1	13.1 ± 1.9	59.7 ± 3.0	12.7 ± 1.8	5.1 ± 1.3	17.8 ± 2.0
Health insurance									
No	199	21.7	29.1 ± 3.9	41.2 ± 3.7	12.1 ± 2.4	53.3 ± 4.9	14.6 ± 2.5	4.0 ± 1.5	18.6 ± 3.0
Yes	719	78.3	22.0 ± 2.6*	47.1 ± 2.2	10.7 ± 1.5	57.9 ± 2.1	10.3 ± 1.5*	4.3 ± 0.8	14.6 ± 1.9
*Mother characteristics*									
Age (years)									
15 to 24	332	36.2	25.6 ± 3.6	42.8 ± 3.0	10.5 ± 2.2	53.3 ± 3.3	13.3 ± 2.6	4.5 ± 1.3	17.8 ± 3.4
25 to 29	274	29.8	21.5 ± 3.9	49.6 ± 3.0***	11.7 ± 1.8	61.3 ± 2.9**	9.1 ± 2.4	5.8 ± 1.4	15.0 ± 3.2
30 or older	312	34.0	23.1 ± 1.8	45.8 ± 2.4	10.9 ± 1.6	56.7 ± 2.8	10.9 ± 1.4	2.6 ± 0.6°	13.5 ± 1.5
Marital status									
Non-union	120	13.1	27.5 ± 4.3	48.3 ± 7.0	16.7 ± 6.6	65.0 ± 3.9	10.0 ± 2.6	11.7 ± 4.7	21.7 ± 3.6
In union	798	86.9	22.9 ± 2.5	45.5 ± 2.5	10.2 ± 1.0	55.6 ± 2.6*	11.4 ± 1.8	3.1 ± 0.4	14.5 ± 1.9*
Schooling									
Elementary or none	156	17.0	28.2 ± 3.8~~	54.5 ± 2.9~~~	9.6 ± 2.9	64.1 ± 3.7~~	16.7 ± 4.0~	3.8 ± 1.5	20.5 ± 4.7~
Secondary school	395	43.0	27.3 ± 3.8	46.8 ± 3.0*	11.6 ± 1.6	58.5 ± 2.7	13.4 ± 2.1	4.8 ± 1.1	18.2 ± 2.6
High school or higher	367	40.0	17.4 ± 1.9°°	41.1 ± 2.9	10.9 ± 2.0	52.0 ± 3.4	6.5 ± 1.1°°	3.8 ± 1.3	10.4 ± 1.9°
Work status during last week									
Did not work	572	62.3	24.5 ± 2.9	44.6 ± 2.7	10.1 ± 1.6	54.7 ± 2.8	11.4 ± 1.7	4.2 ± 0.7	15.6 ± 1.9
Worked	346	37.7	22.0 ± 3.0	48.0 ± 2.0	12.4 ± 1.8	60.4 ± 2.2**	11.0 ± 2.0	4.3 ± 1.1	15.3 ± 2.6
*Household characteristics*									
Nuclear									
No	145	15.8	27.6 ± 3.7	48.3 ± 5.8	16.6 ± 5.7	64.8 ± 3.6	8.3 ± 2.2	11.7 ± 3.9	20.0 ± 3.1
Yes	773	84.2	22.8 ± 2.6	45.4 ± 2.5	10.0 ± 0.9	55.4 ± 2.4**	11.8 ± 1.9	2.8 ± 0.5*	14.6 ± 2.0
Indigenous									
No	651	70.9	23.2 ± 3.3	44.4 ± 2.7	10.1 ± 1.6	54.5 ± 2.7	10.4 ± 2.0	4.3 ± 1.0	14.7 ± 2.5
Yes	267	29.1	24.3 ± 2.5	49.4 ± 3.0	13.1 ± 2.0	62.5 ± 3.9	13.1 ± 1.6	4.1 ± 0.6	17.2 ± 1.9
Socioeconomic status									
Low	306	33.3	33.0 ± 4.5~~~	50.7 ± 3.4	13.1 ± 1.9	63.7 ± 3.1	18.3 ± 3.0~~~	5.9 ± 1.3	24.2 ± 3.5~~~
Medium	307	33.4	21.2 ± 1.8*	40.1 ± 2.7**	10.4 ± 2.1	50.5 ± 3.4**	9.8 ± 1.6*	2.9 ± 1.1	12.7 ± 1.3**
High	305	33.2	16.4 ± 2.5	46.9 ± 2.7°°	9.5 ± 1.1	56.4 ± 3.0	5.6 ± 0.9°	3.9 ± 1.3	9.5 ± 1.6
Overcrowding									
No	711	77.5	23.3 ± 2.4	44.7 ± 2.4	12.0 ± 1.6	56.7 ± 2.9	10.8 ± 1.4	4.6 ± 0.9	15.5 ± 2.0
Yes	207	22.5	24.2 ± 3.9	49.8 ± 2.8	7.7 ± 1.9	57.5 ± 2.8	12.6 ± 2.7	2.9 ± 0.8	15.5 ± 2.9
Beneficiary of social programs									
No	656	71.5	22.6 ± 3.0	44.7 ± 2.6	11.6 ± 1.7	56.3 ± 2.6	10.2 ± 1.6	4.4 ± 1.0	14.6 ± 2.3
Yes	262	28.5	26.0 ± 3.0	48.9 ± 3.2	9.5 ± 2.1	58.4 ± 3.4	13.7 ± 2.3	3.8 ± 1.3	17.6 ± 2.6

Prevalence ± standard error and significance marks for category comparations

*p<0.05,

**p<0.01,

***p<0.001, first vs second category

^~^p<0.05,

^~~^p<0.01,

^~~~^p<0.001, first vs third category

^`^p<0.05,

^``^p<0.01,

^```^p<0.001, first vs forth category

^°^p<0.05,

^°°^p<0.01,

^°°°^p<0.001, second vs third category

^´^p<0.05,

^´´^p<0.01,

^´´´^p<0.001, second vs forth category

^^^p<0.05,

^^^^p<0.01,

^^^^^p<0.001, third vs forth category

### ECD outcomes and their distribution across its proximal determinants

Children aged <12 months had a higher prevalence at risk of neurodevelopmental delay compared to other age groups (Table 1). Male children had a higher prevalence than female children at risk of neurodevelopmental delay (13.8% vs. 7.9%, p<0.001) and a higher prevalence of this condition in conjunction with stunting (5.3% vs. 3.0%, *p* <0.05). Premature children suffered from the highest prevalence at risk of neurodevelopmental delay accompanied with stunting (17.1% ± 3.9). Children with low birth weight had a higher prevalence of stunting than those with normal birth weight (42.6% vs. 21.2%, *p* <0.001), a higher prevalence at risk of neurodevelopment delay (24.8% vs. 9.3%, *p* <0.01) and a greater prevalence of these two conditions simultaneously (16.8% vs. 2.7%, *p* <0.001). The prevalence of chronic undernutrition increased with the number of siblings and was lower among those with health-insurance coverage. Children of mothers who were married or in free union had a lower prevalence of non-normal neurodevelopment (lagging or at risk of delay) and a lower prevalence of this condition with stunting than those born to mother who are neither married or in union (14.5% vs. 21.7%, *p* <0.05). Children of mothers with elementary or no education experienced a higher prevalence of the co-occurrence of non-normal development with stunting compared to children whose mothers had a high-school level or higher education (20.5% vs. 10.4%, *p* <0.05). Children living in nuclear families exhibited a lower prevalence of non-normal neurodevelopment compared to their counterparts (55.4% vs. 64.8%, *p* <0.01), as well as a lower prevalence at risk of developmental delay in conjunction with stunting (2.8% vs. 11.7%, *p* <0.05). Children living in homes with low SES had a higher prevalence of the co-occurrence of stunting and non-normal neurodevelopment compared to children of high SES (24.2% vs. 9.5%, *p* <0.001).

### Post-estimations of covariate-adjusted prevalence from a multiple multinomial logistic regression

After estimating a multiple multinomial logistic regression model including all the analyzed proximal determinants of ECD, we calculated the covariate-adjusted prevalence of stunting, neurodevelopmental outcomes, and their co-occurrence, utilizing predictive margins (Table 2). Compared to females, males had a higher prevalence of stunting, a higher prevalence at risk of neurodevelopmental delay and these two conditions together. Prevalence of the co-occurrence of stunting with being at risk of neurodevelopmental delay was as high as 8.3% (±1.7) among premature children, and higher than that of children with a gestational age ≥39 months (*p* <0,01); this prevalence was also higher among children with lower birth weight than their counterparts (12.3% vs. 2.8%, p <0.01). Compared to children with two or more siblings, those with no siblings had a lower prevalence at risk of neurodevelopmental delay (9.0% vs. 14.8%, p <0.05). Children whose mothers were married or in a free union exhibited a lower prevalence of stunting co-occurring with non-normal neurodevelopment compared with their counterparts (14.9% vs. 40.4%, *p* <0.05). Children whose mothers had elementary education or had not attended school at all experienced a higher prevalence of neurodevelopmental lagging than those whose mothers had a high-school education or more (52.5% vs. 41.1%, *p* <0.01). Children from low SES homes were more likely to experience the co-occurrence of stunting and non-normal neurodevelopment than those from households in the medium and high SES categories (low vs. high: 23.1% vs. 10.5%, p <0.001). The attached supplementary material (S1 Table in S1 File) shows the classical output of multiple multinomial logistic regression model from which we obtained all covariate-adjusted probabilities, along with a brief description of the calculation process for covariate-adjusted prevalence (S1 Appendix material in S1 File).

**Table 2:** Covariate-adjusted prevalence obtained as post-estimations from a multiple multinomial logistic regression model for proximal determinants associated to suboptimal early child development.

	Stunting, %	Neurodevelopment			Stunting and neurodevelopment		
		Lag, %	At risk of delay, %	Lag or risk of delay, %	Lag, %	At risk of delay, %	Lag or risk of delay, %
*Child characteristics*							
Age (months)							
< 12	19.9 ± 2.4	47.1 ± 5.3	19.1 ± 3.6~	66.2 ± 4.9~~~	9.1 ± 2.1	5.5 ± 1.6	14.6 ± 2.0
12 to 23	25.4 ± 2.0*	50.6 ± 2.9	7.9 ± 1.5*	58.5 ± 3.4	13.7 ± 2.2	3.0 ± 0.7	16.7 ± 2.4
24 to 38	23.4 ± 3.8	39.2 ± 2.2°°°	9.4 ± 1.7	48.6 ± 1.8°°	9.4 ± 1.8	4.6 ± 1.3	14.0 ± 2.3
Sex							
Male	26.1 ± 2.1	47.0 ± 2.4	14.6 ± 1.7	61.6 ± 2.3	12.5 ± 1.4	6.0 ± 0.9	18.5 ± 1.7
Female	20.7 ± 2.0*	44.3 ± 2.6	7.3 ± 0.8***	51.6 ± 2.6***	9.7 ± 1.7	2.6 ± 0.6**	12.3 ± 1.7**
Gestational age (months)							
28 to 36	36.8 ± 6.3~~	29.3 ± 4.0~~~	13.2 ± 2.7	42.5 ± 4.0~~	10.9 ± 2.0	8.3 ± 1.7~~	19.3 ± 2.6
37 to 38	27.9 ± 3.8´	51.3 ± 3.8***	7.9 ± 2.9	59.2 ± 3.6**	15.8 ± 4.6	4.3 ± 1.7	20.1 ± 3.6
39 to 40	20.6 ± 2.9	45.6 ± 2.6	12.1 ± 1.6	57.7 ± 2.7	9.7 ± 2.0	3.3 ± 0.7	13.1 ± 2.2
41 to 43	16.9 ± 3.0``	49.5 ± 6.2``	6.9 ± 2.4	56.3 ± 5.4`	11.3 ± 3.3	1.4 ± 1.3``	12.7 ± 3.1``
Low birth weight (<2500g)							
No	21.9 ± 1.4	46.1 ± 2.1	9.4 ± 1.1	55.5 ± 2.2	11.0 ± 1.2	2.8 ± 0.5	13.8 ± 1.3
Yes	34.0 ± 5.7*	44.7 ± 5.4	21.2 ± 4.5*	65.9 ± 4.2*	13.4 ± 3.3	12.3 ± 3.1**	25.7 ± 4.3**
Number of siblings							
None	20.3 ± 2.7	45.6 ± 5.1	9.0 ± 1.6~	54.6 ± 4.9	10.3 ± 1.9	3.4 ± 0.7	13.8 ± 1.8
One	23.4 ± 2.5	46.2 ± 2.9	10.8 ± 1.3	56.9 ± 3.1	11.3 ± 1.8	4.0 ± 0.8	15.3 ± 1.8
Two or more	28.4 ± 3.3	45.6 ± 3.4	14.8 ± 2.4	60.4 ± 3.6	12.3 ± 1.9	6.3 ± 1.4	18.7 ± 2.1
Health insurance							
No	30.1 ± 3.7	39.9 ± 3.4	11.9 ± 2.4	51.7 ± 4.4	14.0 ± 2.3	4.7 ± 1.9	18.7 ± 2.7
Yes	21.7 ± 1.6*	47.5 ± 2.1**	10.7 ± 1.1	58.2 ± 1.9	10.4 ± 1.2	4.1 ± 0.5	14.5 ± 1.3
*Mother characteristics*							
Age (years)							
15 to 24	25.7 ± 2.8	43.9 ± 2.7	10.3 ± 1.8	54.2 ± 3.1	13.7 ± 2.2	4.1 ± 0.9~	17.8 ± 2.5
25 to 29	21.7 ± 3.1	49.2 ± 3.0	12.0 ± 1.6	61.2 ± 2.9	8.9 ± 2.4	6.3 ± 1.3	15.2 ± 2.9
30 or older	22.9 ± 1.8	45.0 ± 2.7	10.9 ± 1.8	55.9 ± 2.7	10.7 ± 1.5	2.6 ± 0.8°	13.3 ± 1.4
Marital status							
Non-union	42.6 ± 10.6	58.2 ± 7.8	6.6 ± 3.4	64.8 ± 8.7	37.8 ± 9.6	2.6 ± 1.0	40.4 ± 9.9
In union	24.2 ± 1.7	45.4 ± 2.7	11.6 ± 1.5	57.0 ± 3.1	10.1 ± 1.2**	4.8 ± 0.9	14.9 ± 1.7*
Schooling							
Elementary or none	26.7 ± 3.7	52.5 ± 3.5~~	9.0 ± 2.8	61.5 ± 3.6	14.5 ± 3.9	4.5 ± 1.6	19.1 ± 4.4
Secondary school	24.8 ± 2.2	47.2 ± 3.1	11.1 ± 1.5	58.3 ± 2.9	12.4 ± 1.5	4.0 ± 0.8	16.3 ± 1.7
High school or higher	20.2 ± 1.6	41.1 ± 3.0	12.3 ± 1.8	53.4 ± 3.5	7.8 ± 1.2°°	4.6 ± 0.9	12.4 ± 1.6
Work status during last week							
Did not work	24.9 ± 2.2	44.4 ± 2.6	10.5 ± 1.6	54.9 ± 2.5	11.2 ± 1.4	4.7 ± 0.8	15.9 ± 1.4
Worked	21.4 ± 2.3	48.2 ± 2.1	12.1 ± 1.5	60.2 ± 2.0*	11.2 ± 1.8	3.6 ± 0.8	14.9 ± 2.1
*Household characteristics*							
Nuclear							
No	33.6 ± 7.7	44.6 ± 7.9	20.9 ± 7.8	65.5 ± 8.7	2.4 ± 0.9	15.6 ± 5.6	18.0 ± 5.8
Yes	25.3 ± 2.5	47.1 ± 2.5	9.1 ± 0.8	56.3 ± 2.4	15.5 ± 2.0***	2.6 ± 0.5*	18.1 ± 1.8
Indigenous							
No	24.0 ± 2.2	45.1 ± 2.5	9.8 ± 1.4	54.9 ± 2.3	11.0 ± 1.8	4.2 ± 0.8	15.2 ± 1.7
Yes	22.4 ± 2.0	47.4 ± 3.0	14.5 ± 2.4	61.9 ± 3.7	11.6 ± 1.2	4.3 ± 1.1	15.9 ± 1.7
Socioeconomic status							
Low	31.8 ± 3.8~~	49.1 ± 3.4	13.4 ± 1.9	62.5 ± 3.0	17.1 ± 2.4~~~	5.9 ± 1.1	23.1 ± 2.6~~~
Medium	21.0 ± 1.9*	40.9 ± 2.8*	10.4 ± 1.9	51.3 ± 3.5**	9.9 ± 1.8*	2.6 ± 0.8**	12.5 ± 1.7***
High	17.4 ± 2.9	47.2 ± 2.8°	9.6 ± 0.9	56.8 ± 3.0	5.9 ± 0.9°	4.4 ± 1.1	10.3 ± 1.5
Overcrowding							
No	24.2 ± 1.5	45.1 ± 2.2	12.2 ± 1.2	57.3 ± 2.4	11.6 ± 1.1	4.6 ± 0.7	16.2 ± 1.3
Yes	21.1 ± 2.9	48.6 ± 3.0	7.2 ± 1.5**	55.8 ± 2.7	10.1 ± 2.0	3.0 ± 0.7	13.1 ± 2.3
Beneficiary of social programs							
No	22.7 ± 2.1	44.7 ± 2.4	11.8 ± 1.4	56.5 ± 2.2	10.3 ± 1.4	4.4 ± 0.7	14.8 ± 1.6
Yes	25.4 ± 2.3	48.9 ± 3.6	9.1 ± 1.9	57.9 ± 4.0	13.3 ± 2.0	3.8 ± 1.4	17.1 ± 2.2

Predictive margins ± standard error and significance marks for category comparations. Total sample size was n = 918, the distributions of covariates are shown in Table 1.

*p<0.05,

**p<0.01,

***p<0.001, first vs second category

^~^p<0.05,

^~~^p<0.01,

^~~~^p<0.001, first vs third category

^`^p<0.05,

^``^p<0.01,

^```^p<0.001, first vs forth category

^°^p<0.05,

^°°^p<0.01,

^°°°^p<0.001, second vs third category

^´^p<0.05,

^´´^p<0.01,

^´´´^p<0.001, second vs forth category

^^^p<0.05,

^^^^p<0.01,

^^^^^p<0.001, third vs forth category

## Discussion

We conducted a study of 918 children ≤3 years old, living in communities suffering from social marginalization in the state of Oaxaca, in Mexico. Based on this sample, we documented a prevalence of nearly 25% for chronic undernutrition, 57% for non-normal or inadequate neurodevelopment, and 15% for both conditions simultaneously. Our study showed that the co-occurrence of stunting and suboptimal neurodevelopment was more prevalent among male children, those born prematurely, those with low birth weight or who lived in homes with low socioeconomic status (SES), and those whose mothers were neither married nor in free union.

The estimated prevalence of chronic undernutrition in this study was higher than figures recently reported by the 2018 100K National Health and Nutrition Survey (ENSANUT by its initials in Spanish) of communities with <100,000 inhabitants (14.9%), and higher than those in the lowest tertile-based stratum of a household economic status index from the ENSANUT 2018 100K (17.5%) [47]. Our study revealed a very high prevalence of inadequate neurodevelopment, with figures significantly greater than evaluation results that caregivers reported by recall in the 2018 100K ENSANUT (16%) [48]. Our findings highlight the gap in neurodevelopmental outcomes and stunting between the socially marginalized communities in our study and locations with <100,000 inhabitants in Mexico. However, this gap in neurodevelopmental outcomes may in part reflect bias in recall reporting. On the other hand, a gap is expected since impoverished conditions undermine the efforts of the disadvantaged to achieve their full potential in both marginalized and smaller communities.

We also found differences in neurodevelopment levels by sex, with male children exhibiting a higher prevalence of indicators for development lag or risk of delay. The results of this study provide empirical support for the presence of a developmental disparity between male and female children and between those born prematurely and those born at full term. Additionally, our findings reaffirm the significance of low birth weight as a crucial predictor of chronic undernutrition and suboptimal neurodevelopment. Another significant finding revealed that the number of siblings was a predictor of being at risk of developmental delay, suggesting competition for resources in households with a greater number of children. Finally, we observed that SES was another predictor of suboptimal neurodevelopment, coinciding with the previous literature [40, 49, 50].

Interventions focused on ECD must prioritize the needs of marginalized communities, where many children present suboptimal neurodevelopment and chronic undernutrition. These results reflect the lack of a legal framework about early childhood that affirms the right of all children, particularly those in marginalized communities, to enjoy optimal ECD. We have demonstrated the need for strategies that aim to provide children with the necessary elements for reaching their full potential and asserting their right to begin their lives on a level playing field. This study also contributes evidence of the pressing need to design interventions focused on the first three years of life, with an emphasis on vulnerable populations. Such interventions should center on reducing the neurodevelopment lag–beginning at birth–that inexorably affects the ECD trajectory of children living in rural, largely indigenous communities. The effects of suboptimal ECD have been shown to last a lifetime.

Fully addressing the determinants of optimal ECD entails the generation of robust evidence to inform the design of public policies. These need to guarantee the five pillars of successful child-rearing practices: quality health and education, space for promoting timely neurological stimulation, food security and the eradication of any form of violence. Sensitive caregivers are a crucial part of the transition towards improved ECD, no less so for those living in impoverished and marginalized conditions. The presence of such caregivers modifies the trajectory of economic and social development in these communities. This, in turn, contributes to the development of human capital, social mobility and reducing disparities in early childhood development [1]. The current COVID-19 pandemic demands multisectoral actions to redefine these transitional strategies and guarantee the rights of children in a context of economic and social recovery. The design of a national strategy to minimize the impact of the global pandemic on vulnerable groups must include the recovery of pathways for meeting the 2030 Sustainable Development Goals, as well as measures to ensure adequate resources, monitoring and evaluation of ECD indicators [51].

Various social policies have been implemented in impoverished contexts in low- and middle-income countries. These policies have sought to guarantee the fundamental rights to health and social protection, promoting equity in childhood living conditions. In Mexico, the “Oportunidades” program stands out [52]. This recently eliminated strategy was implemented by the Mexican government in 1997 initially under the name “Progresa”. It consisted of a conditional cash-transfer scheme aimed at breaking the inter-generational transmission of poverty and promoting the development of human capital in contexts of high marginalization, with a focus on equity. The program featured three components: food, education, and health. Women in participating households received cash transfers, school supplies and food supplements, conditioned on fulfilling certain responsibilities, such as attending medical appointments and remaining in school [53]. Over the years, the program generated favorable outcomes in the areas of health and social development including the following: school retention among young women [54], increased height-for-age [52, 55], improved hemoglobin levels (an indicator for anemia) [55], a decrease in reported diseases [56], less growth retardation, lower prevalence of overweight, as well as greater motor, cognitive, and receptive language development [52]. Several studies have highlighted the narrow window of opportunity for investing in human capital, with interventions occurring at earlier ages shown to be more effective [55, 57]. A positive linear relationship exists between the amount of money invested and favorable outcomes [52]. This underscores the need for greater investment in the development of human capital with a focus on equity and on assuring that children can fully exercise their fundamental rights to health and social protection. However, Mexico has devoted limited resources to social programs specifically aimed at closing the gap in ECD levels resulting from social marginalization and unequal access to health care and education. The results of our study thus represent a useful contribution to discussions concerning the appropriate focus of such efforts.

One study’s results showed the association of preterm birth and low birth weight with ECD, suggesting the need for interventions aimed at achieving quality prenatal care for the most vulnerable households [58]; such interventions have brought about improvements in both birth outcomes [59, 60]. In addition, initiatives that provide food supplements during pregnancy and in the first months of the lives of newborns have proven to be beneficial [61–63]. Another proximal determinant associated with ECD is the number of siblings, underscoring the need for intensive birth-control programs [64] as well as cash-transfer programs that encourage young women to remain in school [53, 54]. Finally, a proximal determinant more difficult to affect directly is SES, which we found to be strongly associated with ECD. Nonetheless, conditional cash-transfer programs such as “Oportunidades” can achieve short-term benefits in improving living conditions in vulnerable households and thus enhance ECD [65].

The association observed between the number of siblings and ECD suggests several hypotheses concerning the mechanisms underlying this relationship. Some researchers have studied the effect of family size and birth order on academic performance, seeking to understand whether this is more the result of a lack of spacing between children or the depletion of parental resources [66]. Future studies should explore this relationship and its causal mechanisms in highly vulnerable communities.

Our study suffered from the limitations common to all cross-sectional analyses. We were unable to establish causal relationships between the proximal determinants analyzed and neurodevelopment and/or chronic undernutrition, finding only associations between these elements. Another limitation concerns voluntary participation, as this can be a source of self-selection bias, with results varying according to the relative levels of development of caregivers and/or parents. Finally, limited sample sizes within certain predictor categories hindered the attainment of higher precision in estimating prevalence.

In sum, our study found important predictors of ECD in the first three years of life regarding two principal indicators: nutritional status and neurodevelopment. Most of these predictors can be modified through social programs and interventions directed at those communities where the prevalence of nutritional and neurodevelopmental problems is high compared to the rest of the country.

## Supporting information

S1 File Proximal determinants of suboptimal early child development during the first three years of life in socially deprived Mexican contexts. S1 Fig (Length or height measurements and age in days compared to WHO growth standard); S1 Table (Multinomial logistic regression model for proximal determinants associated to suboptimal early child development); S1 Appendix material (Multinomial logistic regression model and predictive margins).
(DOCX)
