# Supplementary material for: Proximal determinants of suboptimal early child development during the first three years of life in socially deprived Mexican contexts
Source: PLoS One. 2023 Nov 2;18(11):e0291300. doi: 10.1371/journal.pone.0291300 (PMC10621868; doi:10.1371/journal.pone.0291300)
Supplement: S1 File — S1 Fig (Length or height measurements and age in days compared to WHO growth standard); S1 Table (Multinomial logistic regression model for proximal determinants associated to suboptimal early child development); S1 Appendix material (Multinomial logistic regression model and predictive margins). (DOCX) [file pone.0291300.s001.docx]

**Proximal determinants of suboptimal early child development during the first three years of life in socially deprived Mexican contexts**

**Supplementary material**

Contents

[Fig S1. Length or height measurements and age in days compared to WHO growth standard 2](#_Toc120549605)

[Table S1. Multinomial logistic regression model for proximal determinants associated to suboptimal early child development 3](#_Toc120549606)

Appendix material S1. [Multinomial logistic regression model and predictive margins 5](#_Toc120549607)

## **Fig S1.** Length or height measurements and age in days compared to WHO growth standard

## **Table S1.** Multiple multinomial logistic regression model for proximal determinants associated to suboptimal early child development

|  | Non-stunted with development lag vs non-stunted with normal development | | Non-stunted with risk of development delay vs non-stunted with normal development | | Stunted with normal development vs non-stunted with normal development | | Stunted with development lag vs non-stunted with normal development | | Stunted with risk of development delay vs non-stunted with normal development | |
| --- | --- | --- | --- | --- | --- | --- | --- | --- | --- | --- |
|  | RPR ± SE | P-value | RPR ± SE | P-value | RPR ± SE | P-value | RPR ± SE | P-value | RPR ± SE | P-value |
| *Child characteristics* |  |  |  |  |  |  |  |  |  |  |
| Age (months) |  |  |  |  |  |  |  |  |  |  |
| < 12 | 1 (Ref.) |  | 1 |  | 1 |  | 1 |  | 1 |  |
| 12 to 23 | 0.828 ± 0.220 | 0.479 | 0.291 ± 0.107 | 0.001 | 1.417 ± 0.373 | 0.186 | 1.307 ± 0.479 | 0.465 | 0.437 ± 0.231 | 0.117 |
| 24 to 38 | 0.512 ± 0.126 | 0.006 | 0.219 ± 0.088 | <0.001 | 1.194 ± 0.429 | 0.621 | 0.678 ± 0.295 | 0.373 | 0.535 ± 0.288 | 0.246 |
| Sex |  |  |  |  |  |  |  |  |  |  |
| Male | 1 (Ref.) |  | 1 |  | 1 |  | 1 |  | 1 |  |
| Female | 0.755 ± 0.094 | 0.023 | 0.403 ± 0.103 | <0.001 | 0.830 ± 0.178 | 0.383 | 0.572 ± 0.129 | 0.013 | 0.266 ± 0.103 | 0.001 |
| Gestational age (months) |  |  |  |  |  |  |  |  |  |  |
| 28 to 36 | 1 (Ref.) |  | 1 |  | 1 |  | 1 |  | 1 |  |
| 37 to 38 | 2.399 ± 0.710 | 0.003 | 0.898 ± 0.773 | 0.901 | 0.527 ± 0.286 | 0.238 | 1.790 ± 0.852 | 0.221 | 0.588 ± 0.322 | 0.332 |
| 39 to 40 | 2.296 ± 0.640 | 0.003 | 2.138 ± 1.171 | 0.165 | 0.480 ± 0.204 | 0.084 | 1.009 ± 0.368 | 0.981 | 0.417 ± 0.173 | 0.035 |
| 41 to 43 | 2.130 ± 0.758 | 0.034 | 1.121 ± 0.701 | 0.855 | 0.232 ± 0.145 | 0.020 | 1.016 ± 0.284 | 0.956 | 0.144 ± 0.157 | 0.076 |
| Low birth weight (<2500g) | |  |  |  |  |  |  |  |  |  |
| No | 1 (Ref.) |  | 1 |  | 1 |  | 1 |  | 1 |  |
| Yes | 1.307 ± 0.365 | 0.337 | 2.070 ± 0.924 | 0.103 | 1.526 ± 0.639 | 0.313 | 1.828 ± 0.607 | 0.069 | 7.933 ± 3.876 | <0.001 |
| Number of siblings |  |  |  |  |  |  |  |  |  |  |
| None | 1 (Ref.) |  | 1 |  | 1 |  | 1 |  | 1 |  |
| One | 1.107 ± 0.352 | 0.749 | 1.395 ± 0.462 | 0.314 | 1.400 ± 0.408 | 0.248 | 1.241 ± 0.445 | 0.546 | 1.343 ± 0.414 | 0.339 |
| Two or more | 1.251 ± 0.497 | 0.573 | 2.090 ± 0.808 | 0.056 | 2.034 ± 1.008 | 0.152 | 1.635 ± 0.618 | 0.193 | 2.846 ± 1.522 | 0.050 |
| Health insurance |  |  |  |  |  |  |  |  |  |  |
| No | 1 (Ref.) |  | 1 |  | 1 |  | 1 |  | 1 |  |
| Yes | 1.565 ± 0.305 | 0.021 | 0.992 ± 0.335 | 0.981 | 0.663 ± 0.188 | 0.148 | 0.770 ± 0.181 | 0.266 | 0.896 ± 0.532 | 0.854 |
| *Mother characteristics* |  |  |  |  |  |  |  |  |  |  |
| Age (years) |  |  |  |  |  |  |  |  |  |  |
| 15 to 24 | 1 (Ref.) |  | 1 |  | 1 |  | 1 |  | 1 |  |
| 25 to 29 | 1.600 ± 0.217 | 0.001 | 1.093 ± 0.384 | 0.801 | 0.950 ± 0.355 | 0.891 | 0.752 ± 0.261 | 0.412 | 1.958 ± 0.917 | 0.151 |
| 30 or older | 1.260 ± 0.284 | 0.306 | 1.499 ± 0.508 | 0.232 | 1.325 ± 0.498 | 0.453 | 0.844 ± 0.244 | 0.557 | 0.669 ± 0.173 | 0.121 |
| Marital status |  |  |  |  |  |  |  |  |  |  |
| Non-union | 1 (Ref.) |  | 1 |  | 1 |  | 1 |  | 1 |  |
| In union | 1.824 ± 1.161 | 0.345 | 1.704 ± 1.928 | 0.638 | 4.542 ± 4.173 | 0.100 | 0.207 ± 0.122 | 0.007 | 2.405 ± 2.302 | 0.359 |
| Schooling |  |  |  |  |  |  |  |  |  |  |
| Elementary or none | 1 (Ref.) |  | 1 |  | 1 |  | 1 |  | 1 |  |
| Middle | 0.850 ± 0.144 | 0.339 | 1.530 ± 0.706 | 0.356 | 1.033 ± 0.461 | 0.943 | 0.783 ± 0.259 | 0.461 | 0.793 ± 0.461 | 0.690 |
| Highschool or higher | 0.689 ± 0.131 | 0.050 | 1.399 ± 0.696 | 0.500 | 0.798 ± 0.353 | 0.610 | 0.411 ± 0.141 | 0.01 | 0.786 ± 0.472 | 0.688 |
| Work status during last week | |  |  |  |  |  |  |  |  |  |
| Did not work | 1 (Ref.) |  | 1 |  | 1 |  | 1 |  | 1 |  |
| Worked | 1.226 ± 0.153 | 0.103 | 1.634 ± 0.487 | 0.099 | 0.781 ± 0.232 | 0.406 | 1.097 ± 0.256 | 0.692 | 0.808 ± 0.319 | 0.590 |
| *Household characteristics* |  |  |  |  |  |  |  |  |  |  |
| Nuclear |  |  |  |  |  |  |  |  |  |  |
| No | 1 (Ref.) |  | 1 |  | 1 |  | 1 |  | 1 |  |
| Yes | 0.360 ± 0.207 | 0.075 | 0.580 ± 0.616 | 0.608 | 0.208 ± 0.165 | 0.048 | 4.010 ± 2.830 | 0.049 | 0.054 ± 0.057 | 0.006 |
| Indigenous |  |  |  |  |  |  |  |  |  |  |
| No | 1 (Ref.) |  | 1 |  | 1 |  | 1 |  | 1 |  |
| Yes | 1.230 ± 0.264 | 0.334 | 2.214 ± 0.742 | 0.018 | 0.852 ± 0.212 | 0.519 | 1.223 ± 0.330 | 0.456 | 1.217 ± 0.659 | 0.716 |
| Socioeconomic status |  |  |  |  |  |  |  |  |  |  |
| Low | 1 (Ref.) |  | 1 |  | 1 |  | 1 |  | 1 |  |
| Medium | 0.675 ± 0.143 | 0.064 | 0.736 ± 0.276 | 0.415 | 0.666 ± 0.217 | 0.212 | 0.392 ± 0.117 | 0.002 | 0.254 ± 0.098 | <0.001 |
| High | 1.019 ± 0.187 | 0.917 | 0.533 ± 0.179 | 0.061 | 0.611 ± 0.229 | 0.188 | 0.256 ± 0.066 | <0.001 | 0.531 ± 0.249 | 0.177 |
| Overcrowding |  |  |  |  |  |  |  |  |  |  |
| No | 1 (Ref.) |  | 1 |  | 1 |  | 1 |  | 1 |  |
| Yes | 1.097 ± 0.175 | 0.560 | 0.505 ± 0.183 | 0.059 | 0.928 ± 0.228 | 0.761 | 0.813 ± 0.171 | 0.326 | 0.567 ± 0.234 | 0.170 |
| Beneficiary of social programs |  |  |  |  |  |  |  |  |  |  |
| No | 1 (Ref.) |  | 1 |  | 1 |  | 1 |  | 1 |  |
| Yes | 1.092 ± 0.214 | 0.653 | 0.745 ± 0.282 | 0.437 | 1.096 ± 0.269 | 0.708 | 1.385 ± 0.366 | 0.218 | 0.882 ± 0.513 | 0.829 |
| Refrence RP | 0.591 ± 0.297 | 0.295 | 0.197 ± 0.172 | 0.063 | 0.570 ± 0.234 | 0.17 | 1.152 ± 0.858 | 0.849 | 3.263 ± 4.579 | 0.399 |

RPR: Relative probability ratio; RP: Relative probability. Estimates are PRP ± standard error, except for the last row in which estimates are RP ± standard error.

## **Appendix material S1.** Multiple multinomial logistic regression model and predictive margins

We specified a multiple multinomial logistic regression model with six outcome categories $\left( j=1,..,6 \right)$and fifteen covariates $(k=1,..,15)$.

The model equations express the log of the relative probability of outcome $j=2,\ldots,6$ with respect to outcome $j=1$ (base category) as a function of a linear predictor $\mathbf{x}_{i}'{\hat{\boldsymbol{\beta}}}_{j}$ where ${\hat{\boldsymbol{\beta}}}_{j}$ is the vector of estimated model coefficients and $\mathbf{x}_{i}^{'}=\left[ 1,x_{1i},x_{2i},\ldots,x_{15i} \right]$ is a vector of covariate values (including the constant) for observation $i=1,..,n$ (Equation 1). For each observation, estimated outcome probabilities add up to 1 (Equation 2).

Equation 1. $\ln\left( \frac{\hat{\pi}_{ji}}{\hat{\pi}_{1i}} \right)=\mathbf{x}_{i}'{\hat{\boldsymbol{\beta}}}_{j}$ $j=2,\ldots,6$ $i=1,..,n$

Equation 2. $\sum_{j=1}^{6} \hat{\pi}_{ji}=1$ $i=1,..,n$

For any given observation, the estimated relative probabilities with respect to the base outcome category results from exponentiating the linear predictor (Equation 3). Using this equation along with the restriction in equation 2, estimated probabilities can be calculated for each outcome category and for a set of covariate values $\mathbf{x}_{i}$ (Equations 4 and 5).

Equation 3. $\frac{\hat{\pi}_{ji}}{\hat{\pi}_{1i}}=e^{\mathbf{x}_{i}'{\hat{\boldsymbol{\beta}}}_{j}}$

Equation 4. $\hat{\pi}_{ji}=\frac{e^{\mathbf{x}_{i}'{\hat{\boldsymbol{\beta}}}_{j}}}{1+\sum_{j=2}^{6} e^{\mathbf{x}_{i}'{\hat{\boldsymbol{\beta}}}_{j}}}$ for $j=2,..,6$

Equation 5. $\hat{\pi}_{1i}=\frac{1}{1+\sum_{j=2}^{6} e^{\mathbf{x}_{i}'{\hat{\boldsymbol{\beta}}}_{j}}}$ for $j=1$

For any categorical predictor, relative probability ratios with respect to a reference category can be obtained by exponentiating model coefficients. For example, if there is a predictor indicator variable of female (=1 for females, =0 for males), and its coefficient in the last equation were equal to -1.22, then exp(-1.22)= 0.295 is an estimated relative probability ratio of female vs males. That is, female participants had a relative probability of outcome 6 vs outcome 1 which was 70.5% lower compared to male participants, alternatively, we could say that the relative probability of outcome 6 vs outcome 1 in female participants corresponded to a fraction of 0.295 of the one observed in male participants. Relative probabilities (Equation 3) can be interpreted as the odds of outcome $j$ vs the base outcome category, and relative probability ratios as specific odds ratios (the ratio of the odds of outcome category $j$ vs the base outcome category: ${OR}_{j1}$). Using our previous example, the odds of outcome 6 vs outcome 1 in female participants were 70.5% lower compared to male participants $({OR}_{61}=0.295)$.

*Results as covariate-adjusted probabilities through predictive margins*

The predictive margins approach holds constant the distribution of the other covariates by leaving them in their observed values and changing only the predictor of interest. For each category of the predictor of interest, predictive margins are obtained by averaging probabilities over all observations in the analysis sample. Let the first predictor $k=1$ correspond to a female indicator variable ($x_{1i}=1$ if female, $x_{1i}=0$ if male). Then the predictive margins for the six outcome categories and for female $\left( m_{1j} \right)$ can be calculated as shown in equations 4 and 5.

Equation 4.

$m_{1j}=\frac{1}{n}\sum_{i=1}^{n} \hat{\pi}_{ji}=\frac{1}{n}\sum_{i=1}^{n} \left( \frac{e^{\left[ 1,x_{1i}=1,x_{2i},\ldots,x_{ki} \right]^{'}{\hat{\boldsymbol{\beta}}}_{j}}}{1+\sum_{j=2}^{J} e^{\left[ 1,x_{1i}=1,x_{2i},\ldots,x_{ki} \right]^{'}{\hat{\boldsymbol{\beta}}}_{j}}} \right)$ for $j=2,..,6$

Equation 5.

$m_{1j}=\frac{1}{n}\sum_{i=1}^{n} \hat{\pi}_{1i}=\frac{1}{n}\sum_{i=1}^{n} \left( \frac{1}{1+\sum_{j=2}^{J} e^{\left[ 1,x_{1i}=1,x_{2i},\ldots,x_{ki} \right]^{'}{\hat{\boldsymbol{\beta}}}_{j}}} \right)$ for $j=1$

And for males $(x_{1i}=0$), predictive margins $\left( m_{0j} \right)$ can be calculated as shown in equations 6 and 7

Equation 6.

$m_{0j}=\frac{1}{n}\sum_{i=1}^{n} \hat{\pi}_{ji}=\frac{1}{n}\sum_{i=1}^{n} \left( \frac{e^{\left[ 1,x_{1i}=0,x_{2i},\ldots,x_{ki} \right]^{'}{\hat{\boldsymbol{\beta}}}_{j}}}{1+\sum_{j=2}^{J} e^{\left[ 1,{x_{1i}=0,x}_{2i},\ldots,x_{ki} \right]^{'}{\hat{\boldsymbol{\beta}}}_{j}}} \right)$ for $j=2,..,6$

Equation 7.

$m_{0j}=\frac{1}{n}\sum_{i=1}^{n} \hat{\pi}_{1i}=\frac{1}{n}\sum_{i=1}^{n} \left( \frac{1}{1+\sum_{j=2}^{J} e^{\left[ 1,{x_{1i}=0,x}_{2i},\ldots,x_{ki} \right]^{'}{\hat{\boldsymbol{\beta}}}_{j}}} \right)$ for $j=1$

When we compare $m_{1j}$ with $m_{0j}$, the distribution of the rest of covariates is held constant and the only covariate that changes is $x_{1i}$.

For covariate-adjusted probabilities of the union of outcome categories, we simply added the corresponding predictive margins. For example, for the probability of outcomes 4,5, and 6 as a union (this is the probability of having outcome 4, 5, or 6) we calculated $m_{04}+m_{05}+m_{06}$ for males and $m_{14}+m_{15}+m_{16}$ for females.

All estimated probabilities were multiplied by 100 and described as prevalence (%).
